# Salt and water balance after sweat loss: A study of Bikram yoga

**DOI:** 10.14814/phy2.14647

**Published:** 2020-11-23

**Authors:** Hasan Alrefai, Shannon L. Mathis, Sarah M. Hicks, Aleksandra I. Pivovarova, Gordon G. MacGregor

**Affiliations:** ^1^ Department of Biological Sciences The University of Alabama in Huntsville Huntsville AL USA; ^2^ Department of Kinesiology The University of Alabama in Huntsville Huntsville AL USA; ^3^ Alabama College of Osteopathic Medicine Dothan AL USA; ^4^ Department of Internal Medicine University of Mississippi Medical Center Jackson MS USA; ^5^ YogaLytes Huntsville AL USA

**Keywords:** aldosterone, Bikram yoga, sodium, sweat

## Abstract

Bikram yoga is practiced in a room heated to 105°F with 40% humidity for 90 min. During the class a large volume of water and electrolytes are lost in the sweat, specifically, sodium is lost, the main cation of the extracellular fluid. There is little known about the volume of sweat and the amount of sodium lost in sweat during Bikram yoga or the optimum quantity of fluid required to replace these losses. The participants who took part in this small feasibility study were five females with a mean age of 47.4 ± 4.7 years and 2.6 ± 1.6 years of experience at Bikram yoga. The total body weight, water consumed, serum sodium concentration, serum osmolality, and serum aldosterone levels were all measured before and after a Bikram yoga practice. Sweat sodium chloride concentration and osmolality were measured at the end of the practice. The mean estimated sweat loss was 1.54 ± 0.65 L, while the amount of water consumed during Bikram yoga was 0.38 ± 0.22 L. Even though only 25% of the sweat loss was replenished with water intake during the Bikram yoga class, we did not observe a change in serum sodium levels or serum osmolality. The sweat contained 82 ± 16 mmol/L of sodium chloride for an estimated total of 6.8 ± 2.1 g of sodium chloride lost in the sweat. The serum aldosterone increased 3.5‐fold from before to after Bikram yoga. There was a decrease in the extracellular body fluid compartment of 9.7%. Sweat loss in Bikram yoga predominately produced a volume depletion rather than the dehydration of body fluids. The sweating‐stimulated rise in serum aldosterone levels will lead to increased sodium reabsorption from the kidney tubules and restore the extracellular fluid volume over the next 24 hr.

## Graphical abstract

A large amount of water and salt is lost in sweat during 90 min of Bikram yoga. This fluid loss produced a volume depletion of the extracellular fluid compartment rather than dehydration or disturbance in the body electrolyte levels. The lost fluid will be restored with salt in the diet over the next 24 hr.

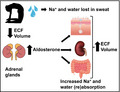

## 1 INTRODUCTION

**FIGURE 1:**
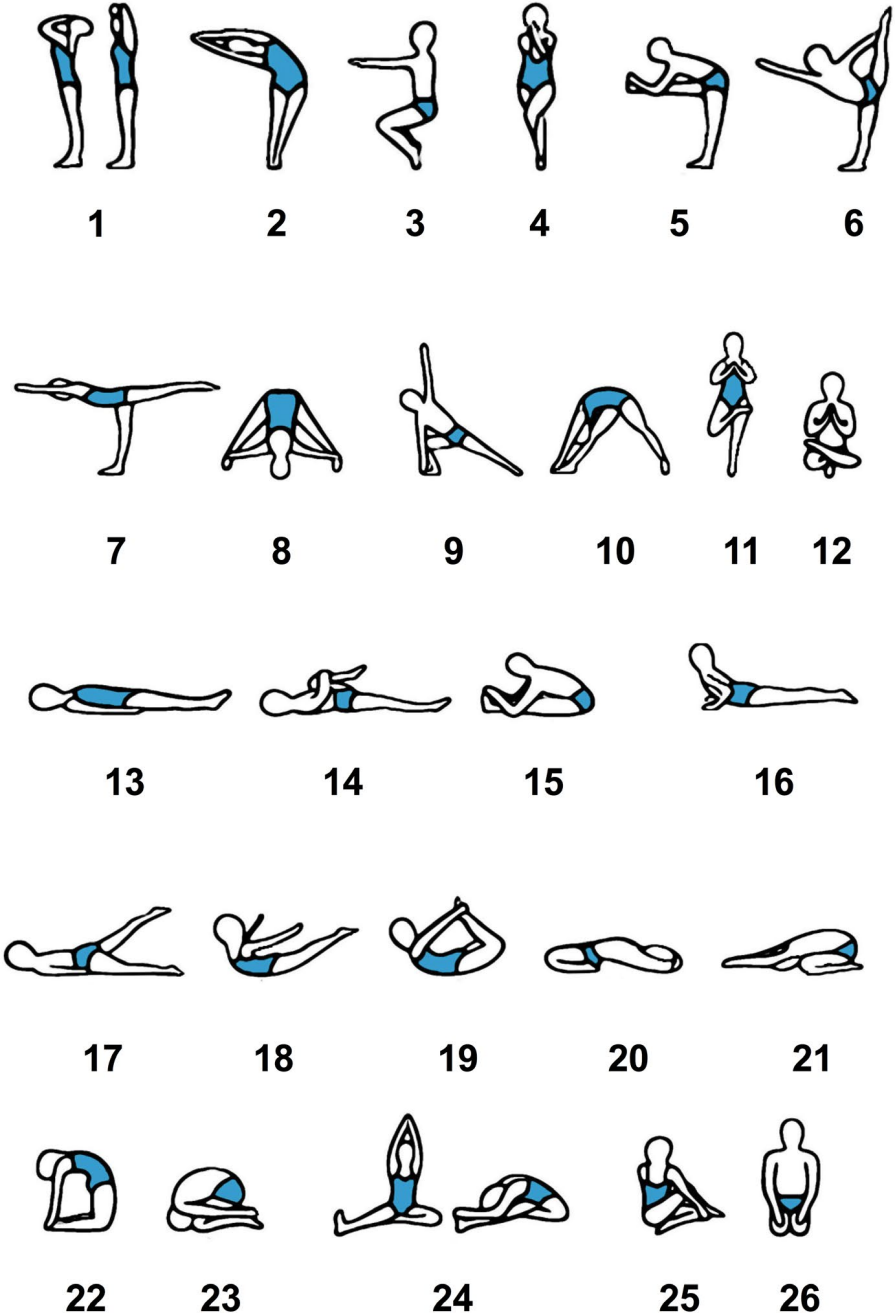
The 26 asana of Bikram yoga. 1. Standing Deep Breathing (Pranayama Series). 2. Half Moon (Ardha Chandrasana) with Hands to Feet (Pada Hasthasana). 3. Awkward Pose (Utkatasana). 4. Eagle Pose (Garurasana). 5. Standing Head to Knee (Dandayamana‐Janushirasana). 6. Standing Bow Pulling Pose (Dandayamana‐Dhanurasana). 7. Balancing Stick (Tuladandasana). 8. Standing Separate Leg Stretching Pose (Dandayamana‐Bibhaktapada‐Paschimotthanasana). 9. Triangle Pose (Trikanasana). 10. Standing Separate Leg Head to Knee Pose (Dandayamana‐Bibhaktapada‐Janushirasana). 11. Tree Pose (Tadasana). 12. Toe Stand (Padangustasana). 13. Dead Body Pose (Savasana). 14. Wind‐Removing Pose (Pavanamuktasana). 15. Sit‐Up. 16. Cobra Pose (Bhujangasana). 17. Locust Pose (Salabhasana). 18. Full Locust Pose (Poorna‐Salabhasana). 19. Bow Pose (Dhanurasana). 20. Fixed Firm Pose (Supta‐Vajrasana). 21. Half Tortoise Pose (Ardha‐Kurmasana). 22. Camel Pose (Ustrasana). 23. Rabbit Pose (Sasangasana). 24. Head to knee Pose (Janushirasana) with Stretching Pose (Paschimotthanasana). 25. Spine‐Twisting Pose (Ardha Matsyendrasana). 26. Blowing in Firm Pose (Kapalbhati in Vajrasana). All the postures from standing pranayama (posture 1) to Standing Separate Leg Head to Knee Pose (posture 10) were repeated twice, while postures 11 and 12 were performed only once on each side of the body. This was followed by a single 2 min savasana (posture 13). Posture 14 was performed twice followed by a single sit‐up (posture 15). Postures 16 to 26 were separated by a minimum of a 20 s savasana, and a single sit‐up (posture 15) was used to transition from savasana to the specific posture between 20 and 26. Posture 25 was performed only once on each side. The final posture and breathing exercise, Blowing in Firm (posture 26) were repeated twice, and the Bikram series concluded with an untimed individual savasana (Choudhury & Reynolds, 1978). This figure and legend have been slightly modified from our first analysis of the Bikram yoga study (Mathis et al., 2020)

Bikram Yoga is a specific selection of 26 Hatha yoga asana (poses), and two breathing exercises, performed in an unchanging, precise sequence with repetition (see details in Figure 1). It was created by Bikram Choudhury in the 1970s and it is considered hot yoga since it is performed in a room heated to 105°F with 40% humidity (Choudhury & Reynolds, 1978). The elevated temperature may increase flexibility and range of motion (Tracy & Hart, 2013) and has even been proposed to add an additional element of focus and meditation that elevates Bikram yoga to a sweaty ritual (Bartholomew, 2018). Although there are significant sweat and electrolyte loss due to the high temperature and humidity in a Bikram Yoga studio (Mathis et al., 2020), little is known about the amount of sodium and water lost in the sweat and the effect that this will have on plasma sodium concentration and body water balance.

In normal body homeostasis, the basic and most critical function of sweat is the simple act of cooling the body when it becomes too hot, either from high environmental temperatures or from exercise (Amatruda & Welt, 1953; Baker, 2019; Robinson & Robinson, 1954). Furthermore, sweat also has a role in skin friction, moisturization and possesses some antibacterial properties (Pasumarty et al., 2011; Schittek et al., 2001; Shiohara et al., 2018). Sweat is hypotonic and mostly water, but contains significant amounts of sodium and chloride (Robinson & Robinson, 1954). Most water‐soluble components of plasma will appear in sweat at a lower concentration than are present in plasma. Sodium, potassium, calcium, magnesium, lactate, glucose, nitrogen (urea, ammonia, creatinine, uric acid), vitamins, iron, manganese, and copper have all been measured in sweat (Baker, 2019; Mitchell & Hamilton, 1949; Robinson & Robinson, 1954). However, potassium, lactate, and urea are frequently measured in sweat at higher concentrations than in plasma (Baker & Wolfe, 2020).

Sweat loss during dehydrating exercise has been shown to increase the sodium concentration of the extracellular fluid compartment (ECF), due to the greater proportion of water rather than electrolyte loss (Costill, 1977; Hew‐Butler et al., 2010; Nose et al., 1988). Interestingly, a replacement of about 50% of the fluid lost in sweat by drinking water, has been shown to prevent the sweating‐induced increase in serum sodium concentrations (Brandenberger et al., 1989; McCubbin et al., 2019; Sanders et al., 1999). This can be explained by the physiological balance of the decrease in total body water (TBW) induced by sweat loss, the amount of sodium lost in the sweat, and the volume of water ingested during exercise.

In Bikram yoga, traditionally, hydration is ad libitum after the fourth asana, Eagle Pose (Garurasana), which occurs about 20–25 min into the 90‐min practice. Following Garurasana, water can be consumed after any asana, but is discouraged before posture 22, Camel Pose (Ustrasana) and 23, Rabbit Pose (Sasangasana) due to the inverted position of the stomach.

Most research on Bikram yoga has focused on the cardiovascular and metabolic effects of the practice (Abel et al., 2012; Hewett et al., 2017; Hunter et al., 2018; Miranda Hurtado et al., 2019; Pate & Buono, 2014). Very little research has been performed on the effect of the profuse sweat loss on serum electrolytes and body fluid balance. Hence, the focus of our research is to quantify sweat loss, sweat composition, and water consumption during Bikram yoga and their effect on body fluid volume and electrolyte levels.

We previously conducted a small feasibility study of fluid and electrolyte balance during Bikram yoga. The first analysis quantified the calcium loss in sweat and investigated the associated body calcium homeostasis (Mathis et al., 2020). In this, the second analysis of the study, we quantify the amount of sodium lost in sweat and its effect on body sodium and water balance during the practice of 90 min of Bikram yoga.

## 2 MATERIALS AND METHODS

### 2.1 Ethical approval

This study was approved by the Institutional Review Boards of UAH (E201666) and ACOM (HS190619EX). All participants were informed of study procedures and signed a written informed consent document prior to study participation. This study conformed to the standards set by the latest version of the Declaration of Helsinki.

### 2.2 Participants and recruitment

Participants eligible for recruitment were females, aged 30–70, who responded to information provided on a flyer posted in the studio of Bikram Hot Yoga of Huntsville (Madison, AL). A total of nine participants were recruited for this small feasibility study. Any person with kidney disease, who use any medication known to affect bone metabolism, and who use blood thinners were excluded from the study. Participants were well hydrated before class as assessed by a serum osmolality measurement. Participants had a mean serum osmolality of 276.4 ± 4.8 mOsmol/kg, where euvolemia is normally considered 275 to ≤295 mOsmol/kg (Thomas et al., 2008). Blood samples were obtained from five subjects and this is the data that we report and analyze.

### 2.3 Sweat collection

The majority of the following methods section is identical to the methods of our analysis of calcium homeostasis during Bikram yoga (Mathis et al., 2020). Nude body weight was measured before and after Bikram yoga (Withings Body Scales, Withings, Inc.). In order to calculate water volume consumed during 90 min of Bikram yoga, each participant's water bottle was weighed before and after yoga using a digital scale (Hario Kitchen Scale, Hario, Inc.). Sweat was collected using a 5 cm by 5 cm square of filter paper (Whatman No. 3, GE Healthcare) that was placed against the thigh during the final yoga posture (Savasana). Sweat was collected by centrifuging the sweat‐loaded filter paper in a Salivette (Sarstedt AG & Co.) tube at 2,500 *g* for 2 min to remove all sweat from the filter paper. The sweat was then centrifuged at 5,000 *g* for 2 min to remove any epithelial cells that may have contaminated the sweat, then stored frozen at −20°C until needed for analysis. Sweat volume loss is calculated from nude body weight assuming that all weight change is considered to be sweat loss and assuming the density of sweat is 1 kg/L. Whole body washdown (WBW) is the gold standard for sweat collection and the measurement of electrolyte loss during sweating (Shirreffs & Maughan, 1997). Sweat from the thigh has been reported to be the best single site for predicting whole body sweat sodium levels determined from WBW measurements (Baker et al., 2009; Patterson et al., 2000). More recently, sodium concentration in sweat from the thigh was found to be similar to sodium measured in whole‐body sweat (Baker et al., 2020), with the highest correlation at moderate humidity, approximately 50%, similar to that in a Bikram yoga studio (Baker et al., 2020). However, sweat samples can be contaminated by surface or sweat pore minerals that may not be completely removed by cleaning (Ely et al., 2011). It was found that only the first sweat sample was contaminated with surface minerals and the second sweat sample taken from the same region 40 min after sweating began did not differ between washed and unwashed skin (Ely et al., 2013). In our study, we did not wash the skin sample area but our sweat sample was taken after 90 min of profuse sweating, removing any effect of skin surface contamination on measured electrolyte levels.

### 2.4 Serum and sweat analyses

A blood sample was collected from the participants immediately before they entered the hot room in the yoga studio and immediately after they left the hot room after 90 min of Bikram yoga. Blood was collected in a serum separator tube and allowed to clot. Serum was collected by centrifuging the blood at 1,500 *g* for 5 min at 4°C. The serum was then aliquoted and stored frozen at −20°C until needed for analysis. Serum sodium was measured using a colorimetric sodium assay (Diazyme Laboratories, Inc.). The Diazyme assay has a good correlation with the ion‐selective electrode method, which is linear between 80 and 180 mmol/L, with a correlation coefficient of 0.98. Serum and sweat osmolality were measured using a Micro Osmometer (Model 3300, Advanced Instruments, Inc). Sweat sodium chloride concentration was measured using a sweat conductivity analyzer (Model 3120, Wescor Inc). The conductivity meter is calibrated in mmol/L of NaCl and the reading obtained represents the concentration of NaCl (mmol/L) in the solution that would exhibit the same conductivity. The meter slightly overestimates the sodium concentration due to potassium and some other electrolytes present in the sweat. Serum aldosterone concentrations were measured using an ELISA assay (E4342, BioVision Inc.). The assays for serum sodium and osmolality were performed in triplicate, and the assay for aldosterone was performed in duplicate and the mean was taken as a single data point. The assay for sweat sodium chloride was a flow‐through conductivity meter and was performed only once. The intra‐assay coefficient of variation (CV%) for the assays was sodium 3.6%, osmolality 0.3%, and aldosterone 11%.

### 2.5 Body volume fluid shift calculations

The TBW (Liters) was calculated for the subjects from anthropometric data using the Watson equation (Watson et al., 1980) and was 29.4 ± 1.6 L which corresponds to 48.9 ± 2.0% of body weight. The extracellular fluid (ECF) compartment was estimated to be 1/3 of total body water and the intracellular fluid compartment (ICF) was estimated to be 66% of the total body fluid volume (Costanzo, 2014). Theoretical changes in body compartment volumes and osmolality were calculated using the water and solute loss measured in sweat. There were two general assumptions. First, intracellular solutes did not shift between the ICF and ECF. Second, all electrolyte and fluid losses came from the ECF. We did not account for the small amount of urine produced or the small amount of sodium lost in the urine during this Bikram yoga study.

### 2.6 Statistical analysis

**FIGURE 2:**
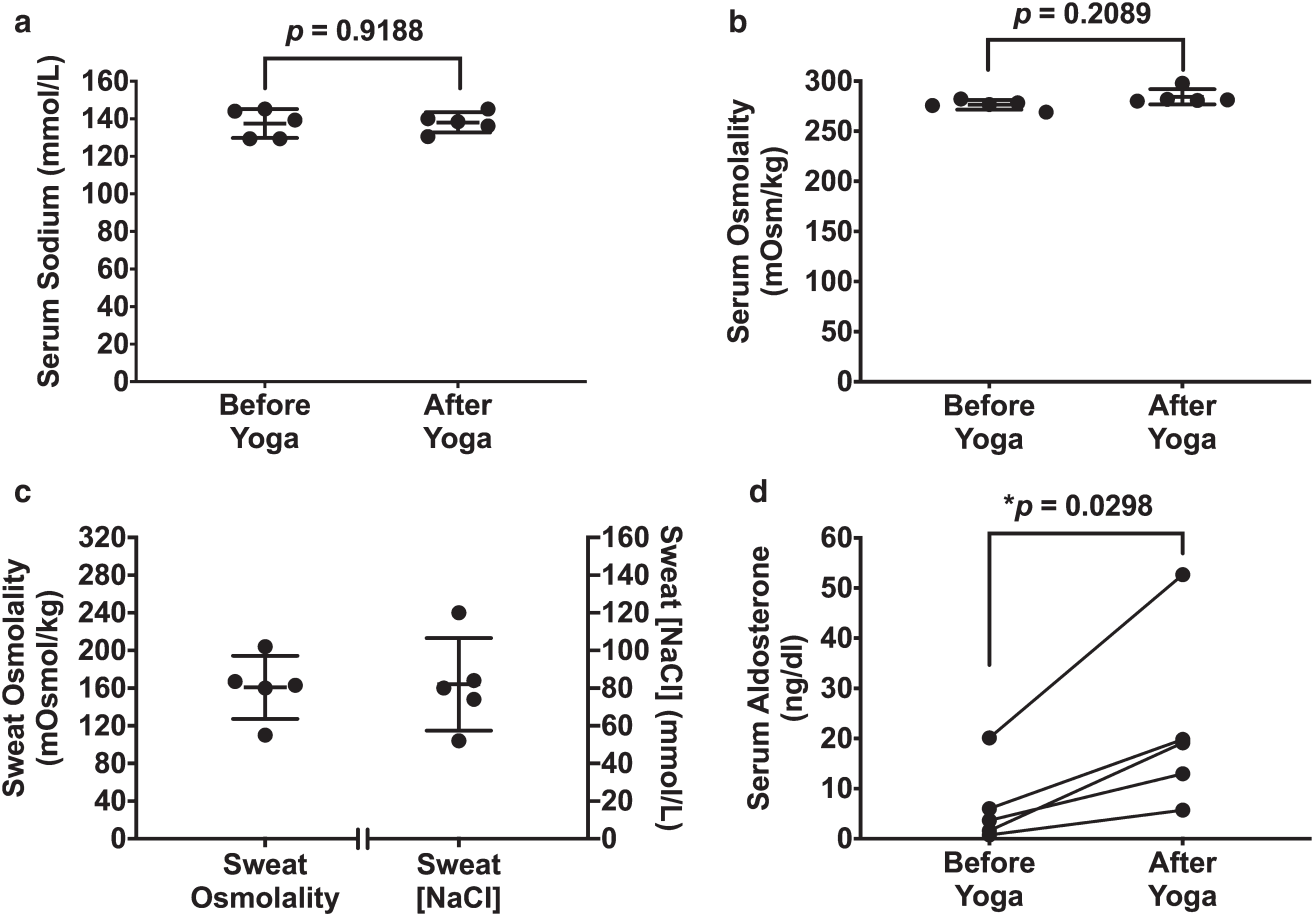
Serum measurements before and after Bikram yoga and sweat properties. (a) Serum sodium levels did not change after 90 min of Bikram yoga. (b) Serum osmolality did not change after Bikram yoga. (c) The osmolality of the sweat was 161 ± 21 mOsmol/kg approximately twice the sweat sodium chloride concentration of 82 ± 16 mmol/L. (d) There was a 3.5‐fold increase in aldosterone levels after 90 min of Bikram yoga

Data are presented as mean ± standard deviation showing individual data points (Figure 2a–c). The serum aldosterone data are shown as individual data points with a connecting line between paired samples (Figure 2d). Pre and posttests were compared using a paired two‐tailed Student's *t* test with an alpha set at 0.05. Data were analyzed and plotted with Prism 8.0 software (version 8.01; GraphPad Software, Inc).

## 3 RESULTS

### 3.1 Participant characteristics

This is the second analysis of our study on electrolyte balance after sweat loss in Bikram yoga. Our first analysis detailed calcium homeostasis and quantified calcium loss in sweat (Mathis et al., 2020). This study was on the same experienced female participants, hence body weights, fluid consumption, and sweat loss are identical to our previous study (Mathis et al., 2020). Subject characteristics are shown in Table 1. Briefly, the participants had a mean age of 47.4 ± 4.7 years (age range 40–53 years) and were experienced female students of Bikram yoga who had attended an average of 4.3 ± 1.3 Bikram yoga classes per week for 2.6 ± 1.6 years. After 90 min of Bikram yoga at 105°F and 40% humidity, participants lost 1.9 ± 0.9% in total body weight from 1.54 ± 0.65 L sweat loss and consumed an average of 0.38 ± 0.22 L of water.

**TABLE 1:** Participant characteristics

Characteristics	Mean ± *SD*
Age (years)	47.4 ± 4.7
Height (cm)	155.5 ± 3.7
Weekly Bikram yoga classes	4.3 ± 1.3
Years of Bikram yoga experience	2.6 ± 1.6
Preyoga nude body weight (kg)	60.4 ± 5.5
Postyoga nude body weight (kg)	59.3 ± 5.4
Sweat lost during yoga (L)	1.54 ± 0.65
Fluid consumed during yoga (L)	0.38 ± 0.22
NaCl concentration of sweat (g/L)	4.8 ± 0.9

### 3.2 Serum and sweat electrolyte levels before and after Bikram yoga

Serum sodium levels did not change and were 137.5 ± 7.7 mmol/L before and 138.1 ± 5.4 mmol/L (*p* = .9188, Figure 2a) after the Bikram yoga session. Serum osmolality was 276.4 ± 4.8 mOsmol/kg, before Bikram yoga and 284.3 ± 7.6 mOsmol/kg, after yoga and also did not change (*p* = .2089, Figure 2b), despite 1.54 ± 0.65 L of sweat loss. The osmolality of the sweat was 161 ± 21 mOsmol/kg, approximately twice the sweat sodium chloride concentration of 82 ± 16 mmol/L (Figure 2c). The estimated total sodium chloride loss in the sweat was 6.8 ± 2.1 g. Serum aldosterone increased from 6.43 ± 7.92 ng/dl before Bikram yoga to 22.06 ± 18.03 ng/dl after Bikram yoga, which is an increase of about a 3.5‐fold (*p* = .0298, Figure 2d).

### 3.3 Body fluid compartments before and after Bikram yoga

**FIGURE 3:**
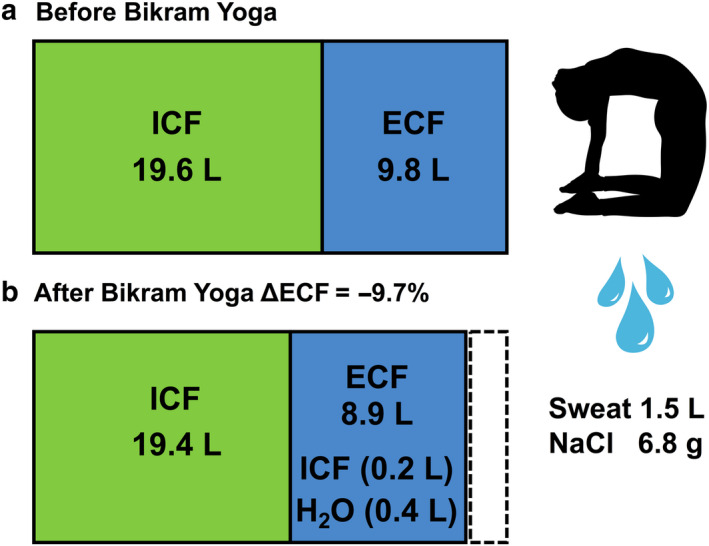
Body fluid compartment changes occur through sweat loss. (a) Total body water was calculated as 29.4 L using the Watson equation. Assuming that 33% of the total body water is located in the ECF, that gives a distribution of ICF 19.6 L and ECF 9.8 L before Bikram Yoga. (b) After Bikram yoga, the participants lost 1.5 L of sweat, containing 160 mOsmol/kg of solutes, and drank approximately 0.4 L of water. This produced an increase in total body osmolality by 3 mOsm/kg and a net shift of 0.2 L from the ICF to the ECF. The dashed box represents the ECF volume lost as sweat. The net result volume contraction of the ECF by 9.7%

The Watson formula was used to calculate TBW in the participants and was 29.4 ± 1.6 L. Assuming an ECF compartment of 33% and an ICF compartment of 66% of TBW and that all sweat solute and water loss is derived from the ECF, this sweat loss would produce an ECF volume decrease of 9.7 ± 4.5% (Figure 3). As the sweat was hypotonic to plasma and was 161 ± 21 mOsmol/kg, whereas the serum osmolality was 276.4 ± 4.8 mOsmol/kg, this measured sweat and solute loss would predict an increase of 3 mOsmol/kg in serum osmolality and a small movement of 0.2 L from the ICF to the ECF (Figure 3).

## 4 DISCUSSION

The aim of this second analysis of our Bikram yoga study was to determine sodium and fluid balance in the body after sweat loss from 90 min of Bikram yoga. We also address the quantity of water necessary for optimal rehydration and the quantity of salt lost in sweat. In our study, participants lost 1.54 ± 0.65 L of sweat during Bikram yoga and only consumed 0.38 ± 0.22 L of water (Mathis et al., 2020). This is considered voluntary hypohydration as participants only replaced 25% of their sweat loss with water. This large mismatch could manifest itself as a decrease in the volume of the ECF (volume depletion) or decrease in TBW (dehydration). We calculated a 9.7% decrease in ECF from body fluid volume and osmolality equations, which was similar to the change in the plasma volume that we previously measured using serum albumin concentrations (Mathis et al., 2020). Hence, in our study, the ECF decreased by a comparable amount as plasma volume, an observation similar to that reported by others (Nose et al., 1988; Senay, 1975, 1979).

### 4.1 The composition of sweat

Sweat is produced from a fluid with the same sodium concentration as interstitial fluid (Bulmer & Forwell, 1956) and sweat ducts have a maximal capacity for reabsorbing sodium, similar to the kidney tubule reabsorbing glucose (Bulmer & Forwell, 1956; Sato, 1977; Thaysen & Schwartz, 1955). As the sweat rate in a single duct increases, the salt reabsorption mechanism will reach its limit and more sodium will start to appear in the surface sweat up to about 100 mmol/L (Harrison, 1974). In our study, after 90 min of Bikram yoga, we measured a sweat sodium chloride concentration of 82 ± 16 mmol/L. The osmolality of the sweat was 161 ± 21 mOsmol/kg, approximately double the sodium concentration, an observation previously reported by others (Buono et al., 2007; Carter et al., 1984). There are other electrolytes and solutes in sweat including calcium, magnesium, potassium, bicarbonate, and lactate that will also contribute slightly to its total osmolality (Baker, 2019). In total, there was a large amount of sodium chloride, 6.8 ± 2.1 g lost in the sweat. We did not observe any changes in the serum sodium concentration as this is dependent on a combination of several variables including, the rate of sweat loss, the concentration of sodium in the sweat, and the quantity of water ingested.

### 4.2 Dehydration versus volume depletion during sweat loss

Dehydration occurs when there has been a decrease in total body water relative to electrolyte content, mainly relative to sodium, the predominant extracellular cation. In dehydration, plasma sodium levels and osmolality will increase, and the majority of the water will be lost from the ICF compartment (Mange et al., 1997). Volume depletion refers to the loss of the extracellular fluid volume, which includes the loss of sodium as well as water. Pure isosmotic volume depletion will decrease the volume of the ECF compartment but will not change in serum sodium concentration or osmolality. A loss of sodium in sweat will, therefore, cause the ECF compartment to contract. The more sodium lost in sweat, the greater the decrease in ECF and plasma volume and more volume depletion. In Bikram yoga, participants lost 1.9% TBW by sweating 1.54 L and only 25% of the sweat loss was replaced by drinking water. There was no change in serum sodium or serum osmolality, suggesting that the sweat loss has resulted in a depletion of extracellular fluid volume rather than dehydration.

### 4.3 Voluntary dehydration

In the classic dessert experiments of Adolph and associates, men marching in the desert heat for 1 hr replaced only 34% of their sweat loss with water (Rothstein et al., 1947). This phenomenon is known as voluntary dehydration, as water is available ad libitum, yet the subjects did not completely replace the water lost in sweat. However, the body fluid deficit was restored later in the day with food during meals. Ingestion of salt with water is required to restore the extracellular fluid volume as any water consumed without salt will simply increase the urine volume via a decrease in circulating ADH levels (Costill et al., 1976). There are many factors to voluntary hypohydration, and serum ADH levels controlling renal water permeability and thirst is just one of them (Greenleaf & Sargent, 1965; Weitzman & Kleeman, 1979). In our study of Bikram yoga, subjects only replaced 25% of their sweat losses with water, usually by drinking 3–4 times during the 90‐min practice (personal observation of G.G.M). We hypothesize that the subjects drank only enough water to normalize their plasma osmolality and sodium levels, and this then satisfied the physiological demand of thirst.

### 4.4 Hydration after Bikram Yoga

Sweat induced changes in body electrolyte levels appear to be minimal after 90 min of Bikram yoga, with no increase in serum sodium or osmolality. As sweat is hypotonic to body fluids, theoretically serum sodium levels should have increased during thermal sweat loss. Whether we observe an increase in serum sodium levels will depend on the amount of sodium lost in the sweat and the volume of water ingested during the sweat loss study. If we model the fluid balance in Bikram yoga using body water volumes (Watson et al., 1980), assuming a 1/3 ECF and 2/3 ICF compartment distribution (Costanzo, 2014) and incorporate the experimentally measured fluid and solute changes, this predicts a small increase in total body and serum osmolality of 3 mOsmol/kg, and a 0.2 L shift of body water from the ICF to the ECF (Figure 3). If an additional 0.3 L of water had been consumed during the yoga practice (for a total of 0.7 L), giving a theoretical fluid replacement of about 45% of the sweat loss, this would have produced no dehydration, no change in body fluid osmolality and no net fluid movement from the ICF to the ECF. If too much water is consumed immediately after a large amount of sweat loss and volume depletion, the electrolytes in the ECF can be diluted and hyponatremia can occur (Noakes et al., 1985). Two instances of hyponatremia have been reported due to excessive rehydration after hot yoga. The first case occurred after the consumption of 3.5 L of water after a beginner's first‐time practice of Bikram yoga, which resulted in a serum sodium level of 120 mmol/L (Reynolds et al., 2012). The second case of hyponatremia was from overhydrating after 90 min of hot yoga in a lactating yogi who consumed about 4 L of fluid, including 3 L of water and 16 oz of kombucha after yoga (Bailowitz et al., 2017). An additional 16 oz of water was consumed on the advice of the yoga studio/health spa upon the subject complaining of a headache later that afternoon. This resulted in a serum sodium level of 126 mmol/L (Bailowitz et al., 2017). Both of these cases were in females with low BMI who weighed under 60 kg. Both subjects had tonic–clonic seizures shortly after admission to the hospital but were successfully treated and recovered with a slow infusion of 3% hypertonic saline solution (Bailowitz et al., 2017; Reynolds et al., 2012).

To efficiently replace the ECF lost in sweat, the appropriate amount of sodium must be included with the water (Costill & Sparks, 1973). If the fluid loss from volume depletion is replaced only by water, this will cause an increase in urine production, due to a decrease in ADH secretion. Salt supplements should be included if this extracellular fluid volume is to be replaced over a shorter time period.

### 4.5 Increased serum aldosterone levels after Bikram yoga

**FIGURE 4:**
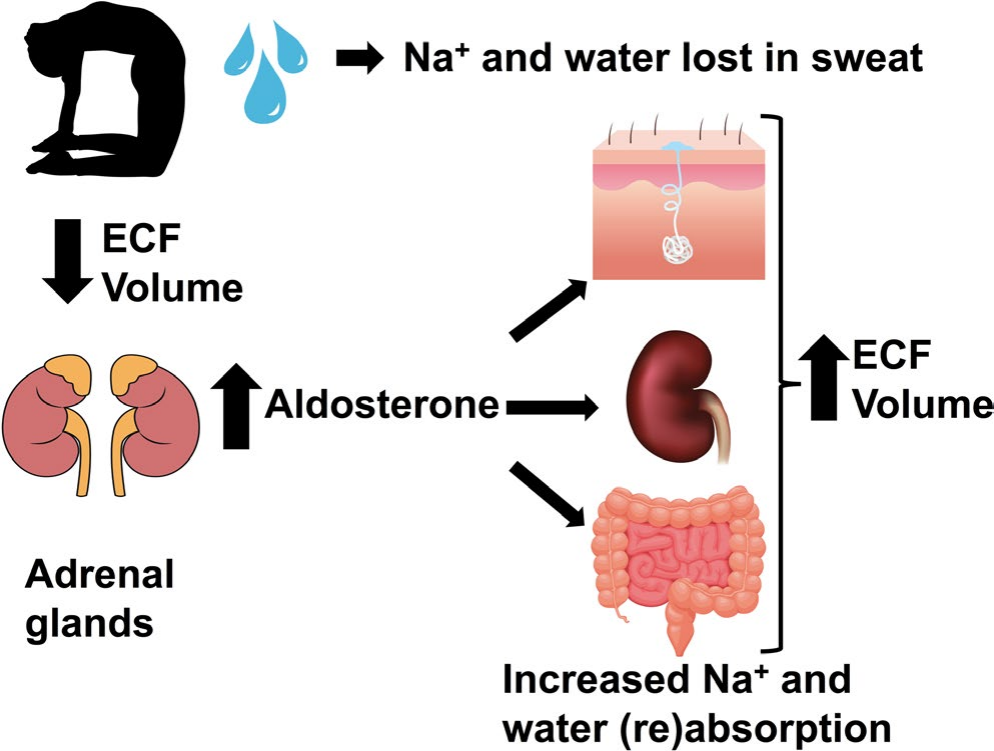
Schematic model of our observations on the physiological effects of sweat loss in Bikram yoga. Water and sodium were lost in the sweat during 90 min of Bikram yoga. This produced a 9.7% decrease in the ECF and effective circulating volume. Renin was released from the kidneys activating the renin–angiotensin–aldosterone system causing an increase in plasma aldosterone. The targets of aldosterone include several sodium transporting epithelia, increasing sodium reabsorption from the gut and from the kidney tubules. The increased sodium reabsorption will increase the osmolality of the blood stimulating the secretion of antidiuretic hormone which will increase water absorption resulting in the restoration of the ECF volume

Sweating‐dependent ECF volume depletion decreases the effective circulating volume and stimulates the renin–angiotensin–aldosterone system (Giebisch & Windhager, 2009a). This promotes sodium retention and volume repletion mainly through the physiological effects of aldosterone stimulating sodium reabsorption in the distal tubules and collecting tubules (Giebisch & Windhager, 2009b). Thermal stress is also sufficient to increase aldosterone release, stimulated by a decrease in blood pressure due to cutaneous vasodilation (Bailey et al., 1972; Hellmann et al., 1956; Kosunen et al., 1976). Studies of exercise in a warm environment where subjects lost about 8.5% of plasma volume with no oral fluid replacement, showed a serum aldosterone increase of about 4‐fold, and this remained elevated for 12 hr after exercise (Costill, Branam, et al., 1976). This produced a decrease in urine production and a decrease in sodium loss in the urine over the next 48 hr as the kidney increased sodium and water reabsorption to restore the ECF volume lost in the sweat (Costill, Branam, et al., 1976). We measured a 3.5‐fold increase in serum aldosterone concentration after 90 min of Bikram yoga. This increase in aldosterone will slowly replenish the 9.7% loss in ECF volume over the next 24 hr by stimulating renal sodium reabsorption from the kidney tubules in an effort to restore plasma volume (Figure 4).

A previous study of hot yoga examined the effect of heat stress on changes in plasma volume (Perrotta et al., 2018). After six consecutive days of hot yoga practice, there was a decrease in plasma volume of 3.5%. Interestingly, a plasma expansion of 5% was observed, 72 hr after the hot yoga intervention ended (Perrotta et al., 2018). Although they did not measure plasma aldosterone levels in this study, this volume expansion may be partially explained by an increase in serum aldosterone levels, similar to what we observed after Bikram yoga.

A small rise in plasma potassium is also an effective stimulus for aldosterone release (Giebisch & Windhager, 2009c). Plasma potassium levels have been shown to increase with exertional heat stress and volume contraction (Costill et al., 1976; Nose et al., 1988); however, we did not measure serum potassium levels in this study. In the presence of increased angiotensin II levels in response to volume depletion, a small rise in plasma potassium can have a more potent effect on aldosterone release from the adrenal cortex (Giebisch & Windhager, 2009c). This would function to maintain renal distal tubule potassium secretion and potassium homeostasis in the presence of volume depletion and a low distal tubule flow rate (Giebisch & Windhager, 2009c). Hence, both a small increase in serum potassium and the volume depletion that we observed may be contributing to the rise in serum aldosterone that we measured after 90 min of Bikram yoga.

### 4.6 Limitations of this study

Due to the small sample size, this study does not have the appropriate statistical power to characterize a small increase in serum sodium levels or serum osmolality. If there is a small approximately 1% change in serum sodium levels and osmolality, which is predicted from the body volume equations and sweat volume and osmolality measurements, this will produce a small shift of body water from the ICF to the ECF. Hence there would be a small element of dehydration in addition to the large component of ECF volume depletion. We have discussed this scenario and it does not change our conclusions. We did not record diuretic, angiotensin‐converting enzyme inhibitor, angiotensin receptor blocker, or mineralocorticoid‐receptor antagonist use in our participants. These drugs may predispose to initial volume depletion or altered aldosterone secretion or action. The sweat sample was collected in the final savasana after 90 min of Bikram yoga. This would ensure it was free from skin contamination; however, the sweat sodium concentration may have been different at an earlier time point. Finally, our sodium loss calculations are based on the thigh being a good representative for total body sodium loss in sweat. Even with the above limitations, our experimental observations are consistent and fall within normal physiological limits.

## 5 CONCLUSIONS

After 90 min of sweating during a Bikram yoga practice, there does not appear to be a major alteration in blood sodium concentration or osmolality, despite a partial replacement of the water lost in sweat. This voluntary hypohydration is sufficient to prevent any changes in body serum sodium levels and osmolality. The water ingested will also minimize the shift of water from the intracellular to the extracellular fluid compartments as seen in dehydration. Sweat loss in Bikram yoga produces an ECF volume depletion rather than dehydration. This loss of ECF will be restored slowly over the next 24 hr by the aldosterone‐regulated retention of sodium and water that is added to body fluids from the diet. It is not necessary to drink large volumes of fluid during Bikram yoga. Replacing about half of the sweat loss with water should prevent any dehydration from occurring. If the ECF volume depletion is needed to be restored more rapidly, then the appropriate amount of salt should be included in the diet.

## CONFLICT OF INTEREST

Gordon G. MacGregor is the owner of YogaLytes Inc, a sweat testing and electrolyte supplement company specializing in hot yoga. The results of the study are presented clearly, honestly, and without fabrication, falsification, or inappropriate data manipulation. HA, SLM, SMH, and AIP, have no conflict of interests.

## AUTHOR CONTRIBUTIONS

GGM, AIP, and SLM conceived and designed the research. GGM and SLM conducted the experiments. HA, SMH, and GGM, analyzed the serum and sweat. GGM analyzed the data. GGM drafted the manuscript, HA, SLM, SMH, and AIP read, edited, and approved the final manuscript.
